# A Study on the Removal of Impurity Elements Silicon and Zinc from Rubidium Chloride by Vacuum Distillation

**DOI:** 10.3390/ma17091960

**Published:** 2024-04-24

**Authors:** Xi Cui, Wenzheng Zhang, Rui Ji, Mingliang Yang, Shichao Wang, Tao Qu

**Affiliations:** 1 Key Laboratory for Nonferrous Vacuum Metallurgy of Yunnan Province, Kunming University of Science and Technology, Kunming 650093, China; cui_xi_1@163.com (X.C.); zhangwz016@163.com (W.Z.); 18225893454@163.com (R.J.); yangml321@163.com (M.Y.); wangshichao0709@163.com (S.W.); 2 National Engineering Research Center of Vacuum Metallurgy, Kunming University of Science and Technology, Kunming 650093, China; 3 Faculty of Metallurgical and Energy Engineering, Kunming University of Science and Technology, Kunming 650093, China; 4 State Key Laboratory of Complex Non-Ferrous Metal Resources Clean Utilization, Kunming University of Science and Technology, Kunming 650093, China

**Keywords:** rubidium chloride, vacuum distillation, vacuum decomposition, chlorides, ab initio molecular dynamics

## Abstract

With the rapid development of high and new technology, rubidium and its compounds show broad application prospect and market demand with their unique characteristics. At present, the production of rubidium metal is mainly prepared by calcium thermal reduction of rubidium chloride. Rubidium metal obtained by reduction requires multi-step vacuum distillation to obtain high-purity rubidium metal. The purity of rubidium metal depends on the purity of the raw material rubidium chloride. Rubidium metal is relatively active and is easy to oxidize and explode in air. Therefore, a method combining vacuum decomposition and vacuum distillation to reduce impurity elements in rubidium chloride from raw materials is proposed in this paper. The experimental results show that under the conditions of pressure of 5–10 Pa, distillation temperature of 823 K and vacuum distillation time of 60 min, the contents of Si and Zn impurities are reduced from 1206 mg/kg and 310 mg/kg to less than 0.1 mg/kg, and the removal rates are 99.99% and 99.97%, respectively. Rubidium chloride has almost no loss, and through one-step vacuum distillation, the impurity elements silicon and zinc can be deeply removed, reducing the flammability and explosiveness, high cost, long process and other problems caused by the subsequent preparation of high-purity rubidium metal.

## 1. Introduction

Rubidium and its compounds have shown broad application prospects and market demand in the defence industry, aerospace, bioengineering, medicine, energy and other high-tech fields due to their unique characteristics such as high and stable radiation frequency, easy ionization, excellent photoelectric characteristics and strong chemical activity [1,2,3,4,5,6]. According to a research report of the United States Geological Survey (USGS) in 2023, the price of 1 g of 99.75% (metal-based) rubidium ampoule is USD 100.80, which is 53% higher than the USD 47.93 in 2003 and 8% higher than the USD 93.40 in 2021. With the rapid development of high technology, the price will increase accordingly while the market demand for high-purity rubidium metal increases. At present, the production of rubidium metal is mainly prepared by calcium thermal reduction of rubidium chloride. The rubidium metal obtained by reduction also needs to undergo multi-step vacuum distillation to obtain high-purity rubidium metal [7]. High-purity rubidium metal has strong chemical activity, which is easy to oxidize and explode in the air. The purity of rubidium metal depends on the purity of the raw materials, and rubidium chloride is the main raw material for the preparation of rubidium metal and other forms of rubidium [8]. Therefore, reducing the content of impurities in the raw material rubidium chloride is of great significance for the preparation of high-purity rubidium metal and meeting the market demand.

As an advanced method, vacuum distillation is mainly based on the vapour pressure difference of each component in the multi-component system, which gives priority to the evaporation of substances with high vapour pressure under the condition of lower atmospheric pressure, and realizes the metallurgical process of component separation and material purification. It has the characteristics of low energy consumption, low pollution and can realize the pollution-free metal resources [9,10,11,12]. Vacuum distillation has been widely used in deep purification of metals, refining of compounds and harmless treatment of hazardous wastes [13,14,15,16]. Therefore, a method for removing impurities from rubidium chloride by vacuum decomposition and vacuum distillation is proposed in this paper. The feasibility of impurity separation was analysed through thermodynamic calculation. The thermal decomposition behaviour of the double salt Rb_2_SiCl_6_ at 723 K and 823 K was simulated by an ab initio molecular dynamics method. The thermodynamic stability of rubidium chloride was studied. The experimental results show that the contents of impurity elements silicon and zinc in rubidium chloride can be effectively reduced by vacuum distillation, and rubidium chloride will not volatilize and decompose.

## 2. Materials and Methods

### 2.1. Materials

The experimental material was rubidium chloride (RbCl, 99% wt%) provided by a company in China. It was produced by the solvent extraction method, in which the extraction and reverse extraction reagents were phenolic reagents and hydrochloric acid, respectively. In the extraction process, silicon (Ⅳ) and zinc (Ⅱ) in the solution are easily extracted into the organic phase by phenolic reagents, and the organic phase is extracted by hydrochloric acid to obtain silicon tetrachloride and zinc chloride [17,18]. At the same time, RbCl will also form a double salt Rb_2_SiCl_6_ with SiCl_4_, so the resulting rubidium chloride is easily mixed with SiCl_4_, Rb_2_SiCl_6_ and ZnCl_2_ [19,20].

**Figure 1:**
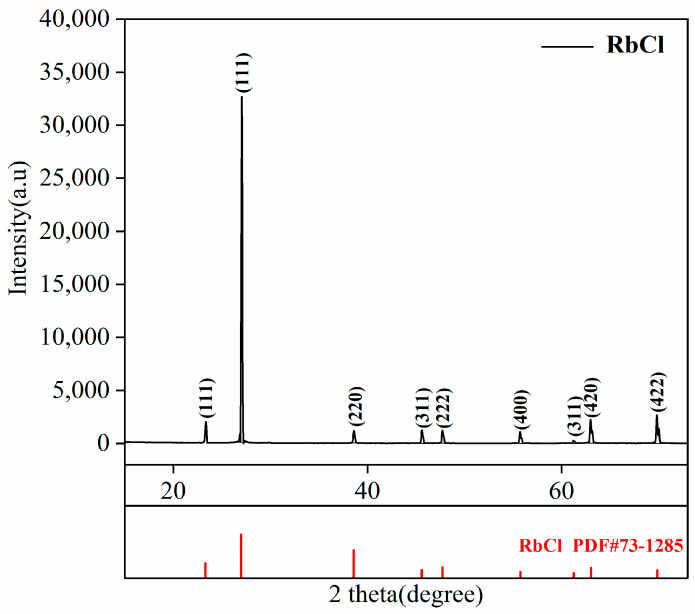
XRD patterns of raw materials.

**Figure 2:**
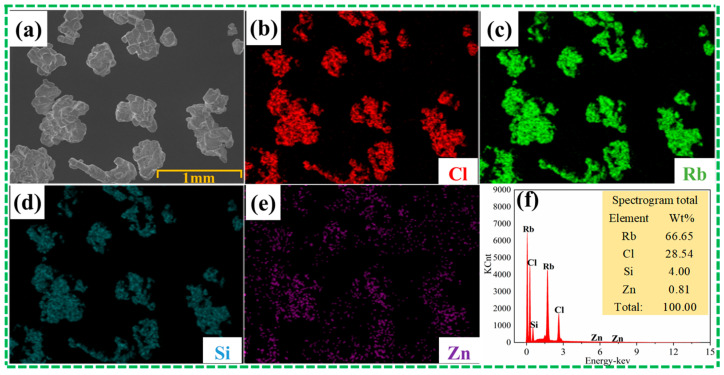
SEM-EDS results of raw materials: (**a**) SEM drawings of raw materials, (**b**–**e**) face scan EDS of image (**a**), (**f**) surface scan result of image (**a**).

Before the experiment, considering that rubidium chloride is hygroscopic and easily absorbs moisture in the air, and that SiCl_4_ is a liquid at room temperature and has a low boiling point of 330 K, we first carried out drying pretreatment of raw materials, dried at 353 K for 8 h, and removed residual moisture and possible SiCl_4_. The physical phase of the raw material is shown in Figure 1, which only contains rubidium chloride, and the content of other elements is low, which is not detected. The morphology and element distribution of the raw material are shown in Figure 2. Rubidium chloride has an irregular shape and uneven particle size. The distribution of the element chlorine coincides with the distribution of rubidium, silicon and zinc and is evenly distributed. After drying at 353 K for 8 h to remove residual water and possible silicon tetrachloride, the remaining silicon exists in the form of the double salt Rb_2_SiCl_6_. After drying, the content of impurity elements silicon and zinc in rubidium chloride is 1206 mg/kg and 310 mg/kg, respectively.

### 2.2. Methods

**Figure 3:**
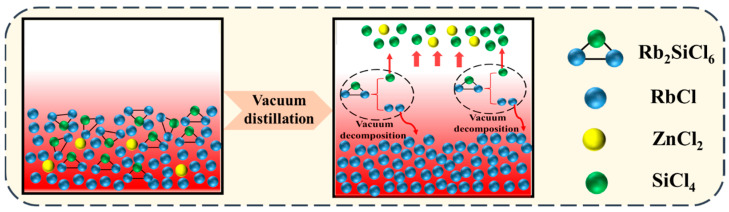
Schematic diagram of vacuum distillation for the removal of the impurity elements silicon and zinc from rubidium chloride.

The process flow of this experiment is shown in Figure 3. ZnCl_2_ was volatilized and removed as a gas phase by vacuum distillation. The double salt Rb_2_SiCl_6_ was decomposed into SiCl_4_ and RbCl in a vacuum, rubidium chloride was retained in the residue and silicon was volatilized in the form of silicon tetrachloride.

**Figure 4:**
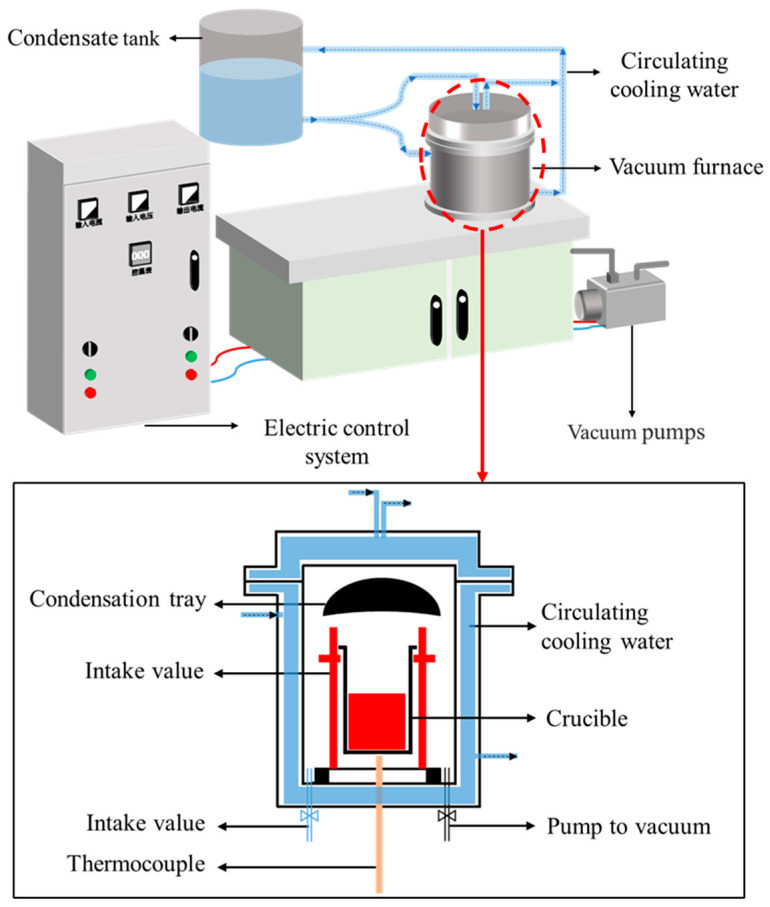
Schematic diagram of the vertical vacuum furnace.

The reaction was carried out in a vertical vacuum furnace, and the schematic diagram of the experimental setup is shown in Figure 4. The equipment mainly includes an electric control system, vacuum furnace, cooling system and vacuum pump. The vacuum furnace is mainly composed of a stainless-steel furnace body, graphite crucible, thermocouple and heat generator.

Firstly, the weighed materials were put into the stainless-steel crucible of the vacuum furnace, the vacuum was drawn, the cooling circulating water was turned on and the heating was carried out at the rate of 10 K per minute until the expected temperature was reached (723 K, 748 K, 773 K, 798 K, 823 K, 848 K). Then, the heating temperature was maintained in the pressure range of 5–10 Pa for 15 min, 30 min, 45 min, 60 min and 75 min, respectively. After holding the temperature for a certain period of time, the heating power was switched off and the samples were cooled under vacuum conditions. When the temperature dropped below 303 K, the vacuum pump was switched off and the residue was removed for analysis. The content of impurity silicon and zinc in the residue was analysed by chemical analysis. The removal rate R of impurity elements was calculated by Equation (1):

(1) $$R=\frac{\left(w_{1}-w_{2}\right)}{w_{1}}$$

where w_1_ and w_2_ are the contents of impurity elements (mg/kg) in raw materials and residues, respectively.

### 2.3. Characterization and Analysis

Inductively coupled plasma mass spectrometry (ICP-MS, ELAN DRC II, PerkinElmer, Waltham, MA, USA) was used to quantitatively analyse the contents of the impurity elements silicon and zinc in the raw materials and residues. The physical phase of the samples was analysed by X-ray diffraction (XRD, X’Pert Pro MPD, Nalytical, Heracles, Almelo, The Netherlands). The samples were characterized by scanning electron microscopy (SEM, TM-3030 Plus, Hitachi, Tokyo, Japan) and energy dispersive spectroscopy (EDS, INCA, Oxford, UK).

## 3. Results and Discussion

### 3.1. Theoretical Analyses

#### 3.1.1. Saturated Vapour Pressure

The saturation vapour pressure difference of metal compounds at different temperatures is an important criterion to determine whether they can be separated by vacuum distillation. In a vacuum, at the same temperature, the saturated vapour pressure of a compound is much higher than the ambient pressure; the higher the saturated vapour pressure of a compound, the easier it is to volatilize; and compounds have a constant vapour pressure at a certain temperature, which can be expressed by Equation (2) [9]:

(2) $$lgp^{*}=AT^{-1}+BlgT+CT+D$$

where p* is the saturation vapour pressure of the pure substance in Pa; T is the thermodynamic temperature in K; and A, B, C and D are evaporation constants.

**Figure 5:**
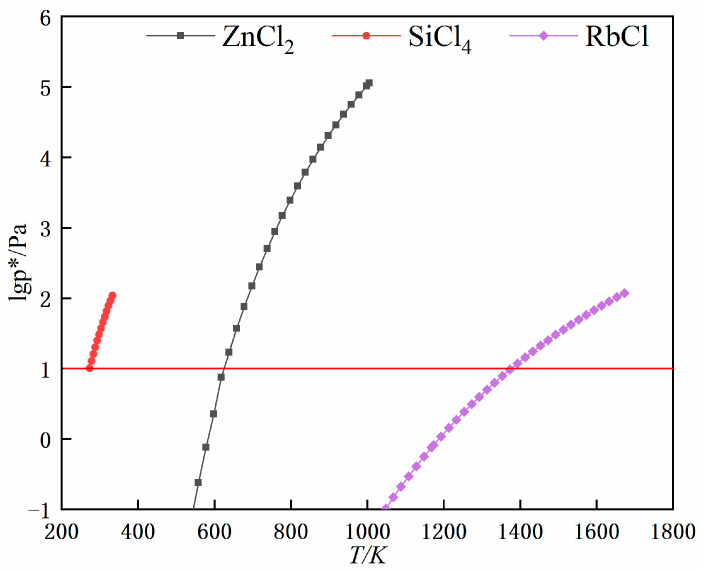
Saturated vapour pressure diagram of various substances.

The compound salt chloride becomes unstable after heating and easily decomposes into simple chlorides at lower temperatures. Therefore, SiCl_4_, ZnCl_2_ and RbCl are used to analyse the thermodynamic behaviour of impurities during vacuum distillation. Figure 5 shows the relationship between the saturated vapour pressure of rubidium chloride, silicon tetrachloride and zinc chloride and the temperature, from which it is clear that the saturation vapour pressure values of SiCl_4_ produced by thermal decomposition of ZnCl_2_ and the complex salts are much larger than those of RbCl at the same temperature. Therefore, by controlling temperature, pressure and experimental steps, zinc chloride in rubidium chloride and silicon tetrachloride produced after the decomposition of double salt can be well removed.

#### 3.1.2. Thermodynamic Calculations

Considering the possibility of volatilization and decomposition of rubidium chloride during distillation, the trend of the reaction can be determined by the Gibbs free energy. The Gibbs free energy change (${△}_{r}G_{T}$) of the reaction can be used as the basis for judging whether the reaction has occurred [9]. The more negative the Gibbs free energy, the more likely the reaction is to occur and vice versa. The Gibbs free energy can be obtained from Equation (3) [9]:

(3) $${△}_{r}G_{T}={△}_{r}G_{T}^{\theta }+RTlnQ$$

where R is the gas constant, T is the temperature and Q is the ratio of the pressure or concentration of the substance before and after the reaction under practical conditions. △_r_$G_{T}^{\theta }$ is calculated by HSC Chemistry 6.0 thermodynamic software.

**Figure 6:**
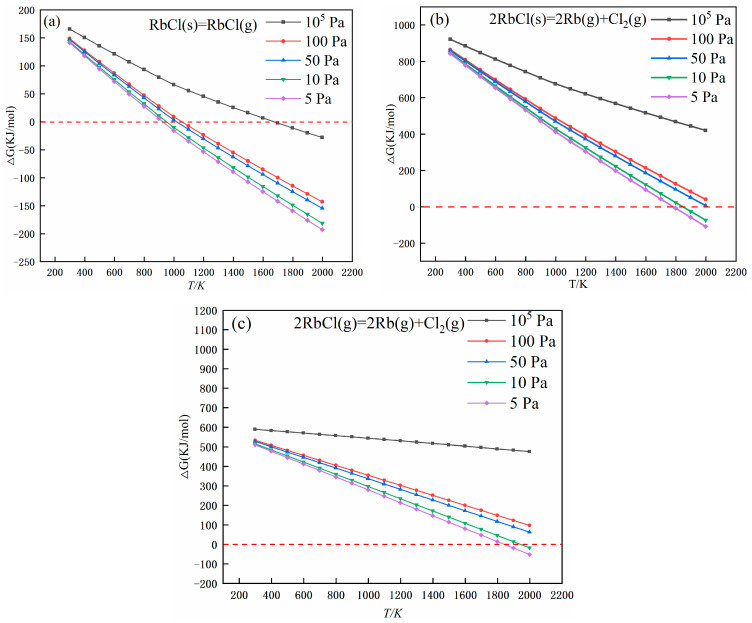
Gibbs free energy changes of rubidium chloride volatilization and decomposition reactions at different pressures: (**a**) the Gibbs free variation of the reaction No.1, (**b**) the Gibbs free variation of the reaction No.1, (**c**) the Gibbs free variation of the reaction No.1.

When the Gibbs free energy of the reaction is less than 0, the reaction can proceed spontaneously. It can be seen from Figure 6 that at 10^5^ Pa, rubidium chloride is relatively stable, and no decomposition reaction occurs. When the temperature reaches 1636 K, rubidium chloride starts to volatilize. As the system pressure decreases, the temperature of the RbCl volatilization reaction and decomposition temperature also decrease gradually, indicating that the vacuum is conducive to the volatilization and decomposition reaction. The lowest volatilization and decomposition temperatures of rubidium chloride were 906 K and 1791 K, respectively, under the condition of 5 Pa. The results of the specific data are shown in Table 1.

**Figure 7:**
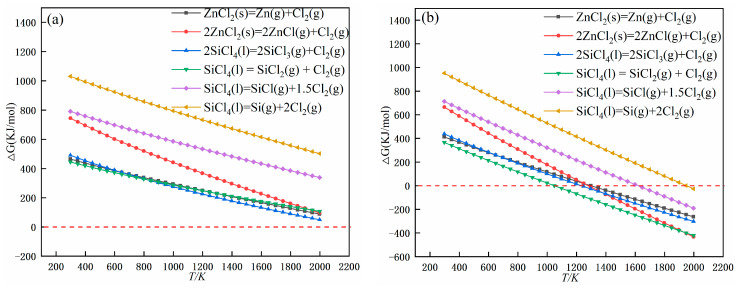
Gibbs energy changes for compound decomposition reactions at (**a**) 10^5^ Pa and (**b**) 5 Pa.

In addition, it is considered that silicon tetrachloride produced by zinc chloride and double salt after thermal decomposition may decompose during the removal process. Therefore, the stability of impurities can also be determined by calculating the initial theoretical decomposition temperature of impurities through Equation (3), and the results are shown in Figure 7. The results show that at 10^5^ Pa, silicon tetrachloride and zinc chloride are very stable. At 5 Pa, the stability of the impurity elements decreases a lot relative to 10^5^ Pa. The lowest decomposition temperatures of SiCl_4_ and ZnCl_2_ are 1049 K and 1280 K, respectively. The results of the specific data collation are shown in Table 2.

In summary, the temperature set must be below 906 K in order to avoid the occurrence of other side reactions during the removal of impurities.

#### 3.1.3. AIMD Simulation Results of Decomposition of Double Salt Rb_2_SiCl_6_

At present, the thermodynamic data of the double salt Rb_2_SiCl_6_ (2RbCl·SiCl_4_) cannot be found in the inorganic thermodynamic databases such as HSC and FactSage. Ab initio molecular dynamics (AIMD) is an effective means to explore the microscopic interaction mechanism of matter and clarify the genetic characteristics of matter [21]. AIMD can explore the mechanism behaviour that is difficult to judge in the experiment from the microscopic perspective and provide certain theoretical guidance for the experiment [22,23,24,25]. The GGA-PBE and NVT integration provides a basic simulation framework, and in practical applications, parameters can be adjusted as needed to optimize simulation performance and accuracy. Therefore, this paper adopts VASP 6.3.0 calculation software calculation based on first principles. GGA-PBE is used to approximate the exchange correlation functional of electron interaction, the energy cutoff is set to 400 eV and the k point is set to 1 × 1 × 1. The plane wave convergence standard of the ion cloth is 1 × 10^−5^ eV/atom, and the atomic convergence standard is 1 × 10^−4^ eV. After structure optimization of the compound salt Rb_2_SiCl_6_, ab initio molecular dynamics simulation was carried out to investigate its decomposition behaviour. Using the NVT ensemble, the molecular dynamics simulation temperature was set to 723 K and 823 K, and the time step and total simulation time were set to 1 fs and 8 ps, respectively.

**Figure 8:**
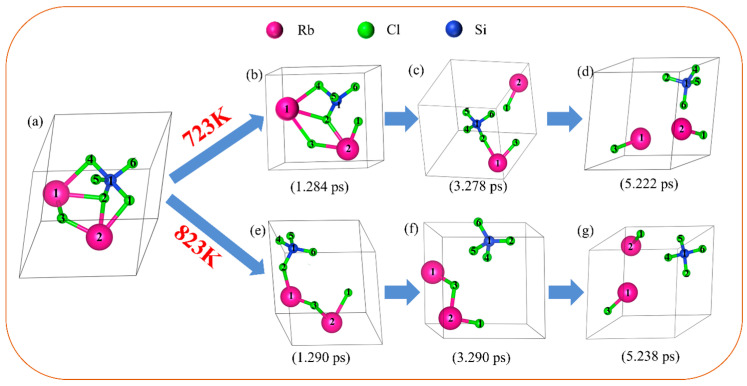
Dynamic simulation results of Rb_2_SiCl_6_ at 723 K and 823 K: (**a**) geometry optimization structure diagram of Rb_2_SiCl_6_, (**b**–**d**) molecular dynamics simulation results of Rb_2_SiCl_6_ at simulated temperature of 723 K and different time (1.284, 3.278, 5.222 ps), (**e**–**g**) molecular dynamics simulation results of Rb2SiCl6 at simulated temperature of 823 K and different time (1.290, 3.290, 5.238 ps).

Figure 8a shows the geometric optimization structure diagram of Rb_2_SiCl_6_ structure, and Figure 8b–d show the molecular dynamics simulation results of Rb_2_SiCl_6_ structure at the simulation temperature of 723 K and different times (1.284, 3.278, 5.222 ps). It can be seen from Figure 8b–d that the structure of Rb_2_SiCl_6_ only changed slightly after 1.284 ps of molecular dynamics simulation, and the Cl1-Si1 bond was broken. After molecular dynamics simulation of 3.278 ps, it can be observed that Cl4-Rb1, Cl2-RB2 and Cl3-Rb2 bonds break and begin to produce RbCl, but the chemical bonds between atoms Rb1 and Si1 and Cl2 are still not broken, and no free SiCl_4_ molecules are produced. After the kinetic simulation of 5.222 ps, it can be observed that the Cl2-Rb1 bond breaks, and two RbCl and one SiCl_4_ appear. Figure 8e–g show the molecular dynamics simulation results of Rb_2_SiCl_6_ at the simulation temperature of 823 K and different times (1.290, 3.290, 5.238 ps). As can be seen from Figure 8e–g, the structure of Rb_2_SiCl_6_ changes greatly after molecular dynamics simulation of 1.290 ps. It can be observed that Cl4-Rb1, Cl1-Si1 and Cl2-Rb2 bonds are broken, but no free SiCl_4_ molecules are generated due to the short simulation time. After molecular dynamics simulation of 3.290 ps, it can be observed that the Cl2-Rb1 bond breaks directly, free SiCl_4_ molecules appear and RbCl and SiCl_4_ structure are separated. After the kinetic simulation of 5.238 ps, it can be observed that the SiCl_4_ molecule still exists statically, the Cl2-Rb1 bond breaks and two RbCl and one SiCl_4_ molecule appear.

Table 3 shows the bond length and population changes of Rb_2_SiCl_6_ before and after kinetic simulation at 723 K and 823 K temperatures. According to the data in Table 3, after geometric structure optimization, the bond lengths of Si1-Cl2, Si1-Cl4, Si1-Cl5 and Si1-Cl6 are 2.0938 Å, 2.24446 Å, 2.09975 Å and 2.12136 Å, respectively, and the populations are 0.5, 0.4, 0.53 and 0.49, respectively. After the kinetic simulation of 5.222 ps at 723 K, the bond lengths of Si1-Cl2, Si1-Cl4, Si1-Cl5 and Si1-Cl6 are 2.06541 Å, 2.0386 Å, 1.9909 Å and 1.97522 Å, respectively, and the populations are as follows: 0.58, 0.6, 0.6 and 0.61. According to the bond length and population, the bond strength of Si1-Cl2, Si1-Cl4, Si1-Cl5 and Si1-Cl6 increased, indicating that the SiCl_4_ structure formed after the decomposition of Rb_2_SiCl_6_ was more stable. After the kinetic simulation of 5.238 ps at 823 K, the bond lengths of Si1-Cl2, Si1-Cl4, Si1-Cl5 and Si1-Cl6 are 2.08553 Å, 2.16396 Å, 2.01125 Å and 2.10425 Å, respectively, and the populations are as follows: 0.53, 0.55, 0.56 and 0.51. It can be seen that the bond strength of Si1-Cl2, Si1-Cl4, Si1-Cl5 and Si1-Cl6 is enhanced, which also indicates that the SiCl4 structure formed after the decomposition of Rb_2_SiCl_6_ is more stable, but the Si1-Cl2, Si1-Cl4, Si1-Cl5 and Si1-Cl6 bonds at 723 K are more stable than those at 823 K.

At 723 K, the bond lengths of Cl3-Rb1 and Cl1-Rb2 are 2.82006 Å and 2.96456 Å, and the populations are 0.15 and 0.04, respectively, after 5.222 ps dynamics simulation. According to the bond length and population, the chemical bond of Cl3-Rb1 and Cl1-Rb2 is strengthened, forming a stable RbCl structure. At 823 K, the bond lengths of Cl3-Rb1 and Cl1-Rb2 are 2.88196 Å and 3.13225 Å, and the populations are 0.11 and 0.02, respectively, after the dynamics simulation of 5.238 ps. It can be seen from the bond length and population that the chemical bond of Cl3-Rb1 and Cl1-Rb2 is strengthened, and a stable RbCl structure is also formed, but the Cl3-Rb1 and Cl1-Rb2 bonds at 723 K are more stable than those at 823 K.

The simulation results of the Rb_2_SiCl_6_ structure at 723 K and 823 K show that Rb_2_SiCl_6_ can decompose at both 723 K and 823 K temperatures. However, when the simulation time is close and the temperature is 823 K, the Cl4-Rb1, Cl1-Rb2 and Cl2-Rb1 bonds connected to SiCl_4_ in the structure of Rb_2_SiCl_6_ can break faster than those at 723 K, resulting in free SiCl_4_ molecules separating RbCl from SiCl_4_. At 723 K, the decomposition of Rb_2_SiCl_6_ is carried out in a distributed step, first to a free RbCl, and then with the increase in simulation time, Rb_2_SiCl_6_ is completely decomposed into two RbCl and one SiCl_4_. At 823 K, Rb_2_SiCl_6_ is decomposed directly into two RbCl and one SiCl_4_ in one step under a short simulation time. It is suggested that increasing the temperature is more conducive to the decomposition reaction of Rb_2_SiCl_6_, which is consistent with the experimental results.

### 3.2. Effect of Distillation Temperature on the Effect of Removing Impurities

**Figure 9:**
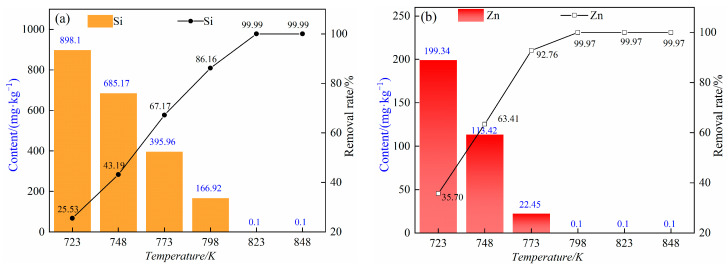
(**a**) Silicon content and removal rate of impurity element under different distillation temperatures with distillation time of 60 min; (**b**) zinc content and removal rate of impurity element under different distillation temperatures with distillation time of 60 min.

Under the pressure range of 5–10 Pa, distillation time of 60 min and distillation temperatures of 723 K, 748 K, 773 K, 798 K, 823 K and 848 K, the content of impurity elements silicon and zinc in rubidium chloride and the removal rate are shown in Figure 9. As can be seen from Figure 9, when the temperature rises from 723 K to 848 K, the content of impurity element silicon gradually decreases, and the removal rates reach 25.53%, 43.19%, 67.17%, 86.19%, 99.99% and 99.99%, respectively. The results show that in the temperature range of 723–848 K, the double salt Rb_2_SiCl_6_ formed by rubidium chloride and silicon tetrachloride decomposes to form silicon tetrachloride and rubidium chloride. The impurity element silicon volatilized into the gas phase in the form of silicon tetrachloride and was removed. With the increase in distillation temperature, the decomposition reaction of compound salt is easier to carry out. In addition, with the increase in distillation temperature, the removal rate of impurity element zinc also increased gradually, which was 35.70%, 63.41%, 92.76%, 99.97%, 99.97% and 99.97%, respectively. When the distillation temperature reached 823 K, continuing to raise the temperature had almost no effect on the removal of impurity elements silicon and zinc; the content of silicon impurity elements and zinc are less than 0.1 mg/kg, a removal rate of nearly 100%.

**Figure 10:**
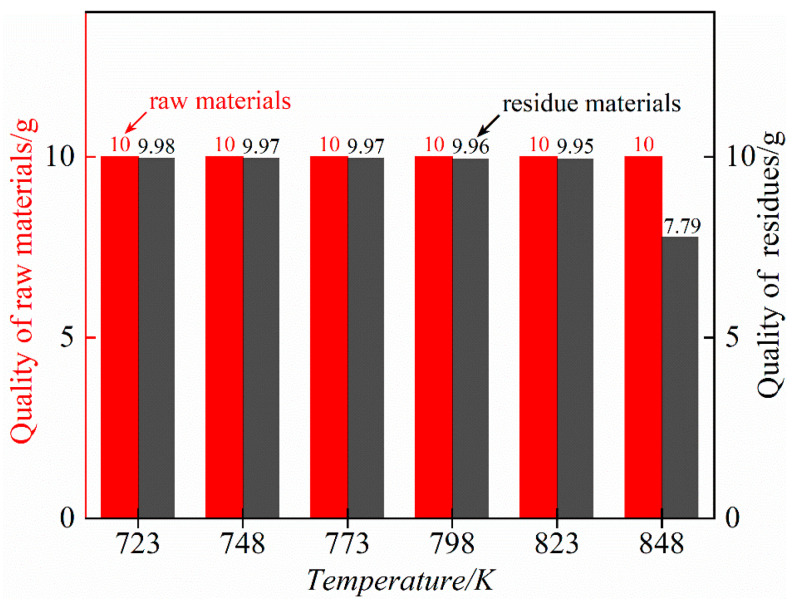
Mass variation of raw material and residue at different distillation temperatures for a distillation time of 60 min.

The mass changes in raw materials and residues at different distillation temperatures are shown in Figure 10. The results showed that in the temperature range of 723–848 K, when the temperature increased to 848 K, the quality of the residue would be obviously reduced, and rubidium chloride began to volatilize. The volatilization temperature of RbCl is at least 50 K lower than that of theoretical calculation. Considering that too low an experimental temperature will lead to a long experimental period, high energy consumption and the impurity elements silicon and zinc cannot be removed completely at the same time, and that too high a temperature will lead to the volatilization of part of the rubidium chloride, we chose 823 K as the optimum distillation temperature for this process.

### 3.3. Effect of Distillation Temperature on the Effect of Removing Impurities

**Figure 11:**
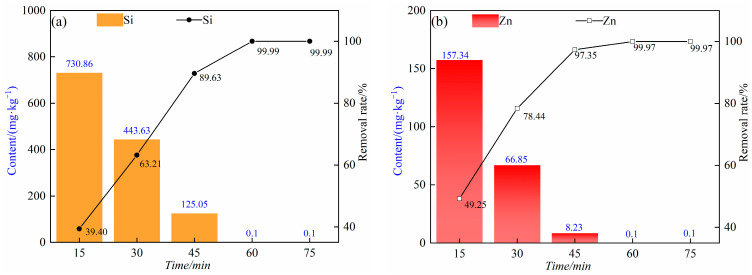
(**a**) The content and removal rate of impurity element silicon at different distillation times at 823 K; (**b**) the content and removal rate of impurity zinc at distillation temperature of 823 K at different distillation times.

Figure 11 shows that the distillation time also has an effect on the removal effect of the impurity elements silicon and zinc. Under the pressure range of 5–10 Pa, distillation temperature of 823 K, and time of 15 min, 30 min, 45 min, 60 min and 75 min, respectively, the removal rate of impurity element silicon gradually increased with the increase in distillation time. They were 39.40%, 63.21%, 89.63%, 99.99% and 99.99%, respectively. In addition, with the increase in distillation time, the content of impurity element zinc also gradually decreased, and the content of impurity element zinc decreased from 157.34 mg/kg to less than 0.1 mg/kg. When the distillation time reaches 60 min, the removal rates of impurity elements silicon and zinc are close to 100%, so the continuous increase in time has little effect on the removal rates of the impurity elements silicon and zinc. Considering the economic cost and impurity removal efficiency, 60 min was chosen as the optimal distillation time.

**Figure 12:**
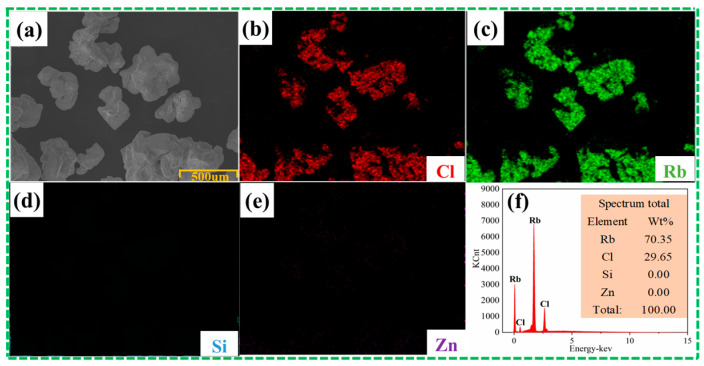
SEM-EDS results of residue under optimal conditions: (**a**) SEM images of residues, (**b**–**e**) face scan EDS diagram of image (**a**), (**f**) surface scan result of image (**a**).

Considering the influence of different distillation temperatures and times on the removal effect of impurity elements silicon and zinc, it can be obtained that under the pressure range of 5–10 Pa, the best process conditions for removing the impurity elements silicon and zinc from rubidium chloride by vacuum distillation are as follows: a distillation temperature of 823 K and a distillation time of 60 min. Under this condition, the impurity elements silicon and zinc can be almost completely removed, the removal rate is close to 100% and the rubidium chloride will not lose. SEM-EDS analysis was carried out on rubidium chloride obtained under the optimal process conditions, and the results were shown in Figure 12. Compared with the EDS results of raw materials, the residue did not contain the impurity elements silicon and zinc.

## 4. Conclusions

1. The thermodynamic calculation and ab initio molecular dynamics results of this study show that the removal of impurity elements silicon and zinc in rubidium chloride is feasible. At a low temperature, the decomposition of the double salt Rb_2_SiCl_6_ is carried out step by step, first decomposed into one RbCl, and then with the increase in time, Rb_2_SiCl_6_ completely decomposed into two RbCl and one SiCl_4_. At higher temperatures, Rb_2_SiCl_6_ directly decomposed into two RbCl and one SiCl_4_, and increasing the temperature is more conducive to the decomposition of Rb_2_SiCl_6_.
2. Under the pressure range of 5–10 Pa and the temperature range of 723~823 K, ZnCl_2_ is volatilized and removed in the form of gas phase, and silicon in the form of the double salt Rb_2_SiCl_6_ will decompose into SiCl_4_ and RbCl; SiCl_4_ is volatilized and removed in the form of gas phase, and RbCl can exist statically in the residue. At temperatures as high as 848 k, rubidium chloride will be volatile.
3. The results of different experimental conditions show that the optimal process parameters for removing impurity elements silicon and zinc in rubidium chloride under the pressure range of 5–10 Pa are as follows: a distillation temperature of 823 K and a distillation time of 60 min. Under such conditions, rubidium chloride will not lose. Through one-step vacuum distillation, the contents of the impurity elements silicon and zinc in rubidium chloride decreased from 1206 mg/kg and 310 mg/kg to less than 0.1 mg/kg and 0.1 mg/kg, respectively, with removal rates of 99.99% and 99.97%. Therefore, the content of the impurity elements silicon and zinc in rubidium chloride is effectively reduced by this process, which lays a foundation for further purification of rubidium chloride by vacuum distillation.

## Figures and Tables

**Table 1:** Theoretical initial reaction temperature of RbCl under different pressures.

No.	Reaction Equation	Theoretical Reaction Temperature/K				
		10^5^ Pa	100 Pa	50 Pa	10 Pa	5 Pa
1	RbCl(s) = RbCl(g)	1636 K	1025 K	1007 K	924 K	906 K
2	2RbCl(s) = 2Rb(g) + Cl_2_(g)	—	—	—	1821 K	1791 K
3	2RbCl(g) = 2Rb(g) + Cl_2_(g)	—	—	—	1900 K	1819 K

**Table 2:** Theoretical decomposition temperatures of compounds at different pressures.

No.	Reaction Equation	Theoretical Reaction Temperature/K	
		10^5^ Pa	5 Pa
1	ZnCl_2_(s) = Zn(g) + Cl_2_(g)	—	1298 K
2	2ZnCl_2_(s) = 2ZnCl(g) + Cl_2_(g)	—	1280 K
3	2SiCl_4_(l) = 2SiCl_3_(g) + Cl_2_(g)	—	1233 K
4	SiCl_4_(l) = SiCl_2_(g) + Cl_2_(g)	—	1049 K
5	SiCl_4_(l) = SiCl(g) + 1.5Cl_2_(g)	—	1623 K
6	SiCl_4_(l) = Si(g) + 2Cl_2_(g)	—	1949 K

**Table 3:** Bond length and population before and after molecular dynamics simulation of Rb_2_SiCl_6_.

Bond	Rb_2_SiCl_6_ System (0 ps)		Rb_2_SiCl_6_ System (723K, 5.222 ps)		Rb_2_SiCl_6_ System (823K, 5.238 ps)	
	Bond Length/Å	Population	Bond Length/Å	Population	Bond Length/Å	Population
Cl2-Rb1	2.11052	0.14	—	—	—	—
Cl3-Rb1			2.82006	0.15	2.88196	0.11
Cl4-Rb1	2.12621	0.2	—	—	—	—
Cl1-Rb2			2.96456	0.04	3.13225	0.02
Si1-Cl2	2.0938	0.5	2.06541	0.58	2.08553	0.53
Si1-Cl4	2.24446	0.4	2.0386	0.6	2.16396	0.55
Si1-Cl5	2.09975	0.53	1.9909	0.6	2.01125	0.56
Si1-Cl6	2.12136	0.49	1.97522	0.61	2.10425	0.51

## Data Availability

The data presented in this study are available upon request from the corresponding author. The data are not publicly available because related studies are ongoing.
